# Deletion of Nardilysin Prevents the Development of Steatohepatitis and Liver Fibrotic Changes

**DOI:** 10.1371/journal.pone.0098017

**Published:** 2014-05-21

**Authors:** Shoko Ishizu-Higashi, Hiroshi Seno, Eiichiro Nishi, Yoshihide Matsumoto, Kozo Ikuta, Motoyuki Tsuda, Yoshito Kimura, Yutaka Takada, Yuto Kimura, Yuki Nakanishi, Keitaro Kanda, Hideyuki Komekado, Tsutomu Chiba

**Affiliations:** 1 Department of Gastroenterology and Hepatology, Kyoto University Graduate School of Medicine, Kyoto, Japan; 2 Department of Cardiovascular Medicine, Kyoto University Graduate School of Medicine, Kyoto, Japan; Institute of Medical Research A Lanari-IDIM, University of Buenos Aires-National Council of Scientific and Technological Research (CONICET), Argentina

## Abstract

Nonalcoholic steatohepatitis (NASH) is an inflammatory form of nonalcoholic fatty liver disease that progresses to liver cirrhosis. It is still unknown how only limited patients with fatty liver develop NASH. Tumor necrosis factor (TNF)-α is one of the key molecules in initiating the vicious circle of inflammations. Nardilysin (N-arginine dibasic convertase; Nrd1), a zinc metalloendopeptidase of the M16 family, enhances ectodomain shedding of TNF-α, resulting in the activation of inflammatory responses. In this study, we aimed to examine the role of Nrd1 in the development of NASH. *Nrd1^+/+^* and *Nrd1^−/−^* mice were fed a control choline-supplemented amino acid-defined (CSAA) diet or a choline-deficient amino acid-defined (CDAA) diet. Fatty deposits were accumulated in the livers of both *Nrd1^+/+^* and *Nrd1^−/−^* mice by the administration of the CSAA or CDAA diets, although the amount of liver triglyceride in *Nrd1^−/−^* mice was lower than that in *Nrd1^+/+^* mice. Serum alanine aminotransferase levels were increased in *Nrd1^+/+^* mice but not in *Nrd1^−/−^* mice fed the CDAA diet. mRNA expression of inflammatory cytokines were decreased in *Nrd1^−/−^* mice than in *Nrd1^+/+^* mice fed the CDAA diet. While TNF-α protein was detected in both *Nrd1^+/+^* and *Nrd1^−/−^* mouse livers fed the CDAA diet, secretion of TNF-α in *Nrd1^−/−^* mice was significantly less than that in *Nrd1^+/+^* mice, indicating the decreased TNF-α shedding in *Nrd1^−/−^* mouse liver. Notably, fibrotic changes of the liver, accompanied by the increase of fibrogenic markers, were observed in *Nrd1^+/+^* mice but not in *Nrd1^−/−^* mice fed the CDAA diet. Similar to the CDAA diet, fibrotic changes were not observed in *Nrd1^−/−^* mice fed a high-fat diet. Thus, deletion of nardilysin prevents the development of diet-induced steatohepatitis and liver fibrogenesis. Nardilysin could be an attractive target for anti-inflammatory therapy against NASH.

## Introduction

Nonalcoholic fatty liver disease (NAFLD) is a condition in which excess fat accumulates in the hepatocytes of patients without a history of alcohol abuse [1]. NAFLD is a hepatic manifestation of metabolic syndromes, such as obesity, type-II diabetes mellitus, and hyperlipidemia. Its prevalence is increasing particularly in the developed countries [1], [2]. Nonalcoholic steatohepatitis (NASH) is a severe form of NAFLD, in which liver inflammation is observed and which progresses to liver fibrosis. A part of NAFLD patients develops NASH that leads to liver fibrosis. However, the exact causes and mechanisms of the development of NASH remain unknown.

Recent investigations have suggested a “multi-hit process” model for the development of NASH [3]. Liver inflammation including NASH can be initiated or enhanced by multiple cytokines secreted mainly by Kupffer cells or macrophages [4]. During liver fibrogenesis, myofibroblasts, that are not present in normal liver, also contribute to liver fibrogenesis through the remodeling of extracellular matrix [5]. In pro-inflammatory cascades, there are several key factors that play a crucial role in initiating or halting inflammation. Tumor necrosis factor (TNF)-α is one of such key molecules, and anti-TNF-α therapies are used widely to treat human inflammatory disorders, such as rheumatoid arthritis and inflammatory bowel diseases [6], [7]. To activate TNF-α, a membrane-bound pro-TNF-α must be appropriately and sufficiently cleaved by the prototypical sheddase, TNF-α-converting enzyme (TACE) [8]. Previously, we showed that nardilysin (N-arginine dibasic convertase; Nrd1), a zinc metalloendopeptidase of the M16 family that ubiquitously localizes in various organs, enhances the shedding of TNF-α through TACE activation [9]–[13]. Nardilysin binds to TACE and directly enhances its catalytic activity [10], [11]. It also promotes the ectodomain shedding of TNF-α, resulting in activation of the TNF-α/nuclear factor-κB pro-inflammatory signaling cascade [12].

In this study, we aimed to elucidate the mechanisms that distinguish NASH from simple liver steatosis. We examined the role of nardilysin, that is known to enhance TNF-α shedding, in the development of steatohepatitis using *Nrd1^+/+^* and *Nrd1^−/−^* mice fed a choline-deficient and amino acid-defined (CDAA) diet and a high-fat diet (HFD), that are used widely to reproduce the natural course of NASH and liver fibrosis in mice as well as in humans.

## Materials and Methods

### Ethics statement

All animal experiments were undertaken in accordance with institutional guidelines. The Review Board of Kyoto University granted ethical approval for this study.

### Animal models

Nardilysin-deficient (*Nrd1^−/−^*) mice (CDB0466K: http://www.cdb.riken.jp/arg/mutant%20mice%20list.html) were previously described [13]. Male *Nrd1^+/+^* and *Nrd1^−/−^* mice with the BL6/CBA background were bred and housed in a temperature- and light-controlled facility with unlimited access to food and water. To induce steatohepatitis and liver fibrotic changes, 10–12-week old male mice were fed a control choline-supplemented amino acid-defined (CSAA) diet or a choline-deficient amino acid-defined (CDAA) diet (Research Diets, New Brunswick, NJ, USA) for 4, 12, or 20 weeks according to the previous reports [14], [15]. As another diet-induced model of steatohepatitis and liver fibrosis, mice were fed a HFD (Oriental Bio Service, Kyoto, Japan) for 20 weeks on the basis of previous studies [16], [17]. Triglyceride levels in the livers were determined with Triglyceride Quantification kit (Abcam, Cambridge, MA, USA) according to the manufacture's protocol. Serum levels of alanine aminotransferase (ALT) were measured using a Transaminase CII-Test Wako kit (Wako Pure Chemical Industries, Osaka, Japan).

### Histological analyses and immunostainings

The liver was resected at various time points, fixed with 4% buffered paraformaldehyde solution, embedded in paraffin, and sectioned into 5-µm thickness. Oil red O (Wako Pure Chemical Industries) staining was performed to confirm fatty deposition. Sirius red (saturated picric acid containing 0.1% Direct Red 80 and 0.1% Fast Green FCF; Sigma-Aldrich, St. Louis, MO, USA) staining was done to visualize collagen deposition. Stained fibrotic areas were measured as percentage area in a representative ×100 high-power field in each mouse using Image J software. For the immunostainings the sections were incubated overnight with the primary antibodies at 4°C, after which the secondary antibodies were added. Kupffer cells or macrophages were stained with rat anti-F4/80 monoclonal antibody (Abcam). TNF-α staining was performed with anti-TNF-α goat polyclonal antibodies (R&D systems, Minneapolis, MN, USA). Activated myofibroblasts were stained with anti-α-smooth muscle actin (SMA) rabbit polyclonal antibody (Abcam). Negative controls were prepared with isotype IgG.

### Real-time quantitative reverse transcription-polymerase chain reaction (qRT-PCR)

Total RNA was extracted using Trizol (Life Technologies, Carlsbad, CA, USA). Single-strand complementary DNA (cDNA) was synthesized using a Transcriptor First Strand cDNA Synthesis kit (Roche Applied Science, Basel, Switzerland). qRT-PCR was performed using SYBR Green I Master (Roche Applied Science) and Light Cycler 480 (Roche Applied Science). Values are expressed as arbitrary units relative to glyceraldehyde 3-phosphate dehydrogenase (GAPDH). The primer sets used were: TNF-α-Forward, CCCTCACACTCAGATCATCTTCT, TNF-α-Reverse, GCTACGACGTGGGCTACAG; interleukin (IL) 6-Forward, TAGTCCTTCCTACCCCAATTTCC, IL6-Reverse, TTGGTCCTTAGCCACTCCTTC; IL1-β-Forward, GCAACTGTTCCTGAACTCAACT, IL1-β-Reverse, ATCTTTTGGGGTCCGTCAACT; CCR2-Forward, ATCCACGGCATACTATCAACATC, CCR2-Reverse, CAAGGCTCACCATCATCGTAG; collagen I-Forward, GCTCCTCTTAGGGGCCACT, collagen I-Reverse, ATTGGGGACCCTTAGGCCAT; collagen IV-Forward, TCCGGGAGAGATTGGTTTCC, collagen IV-Reverse, CTGGCCTATAAGCCCTGGT; tissue inhibitor of metalloproteinase (Timp) 1-Forward, CTTGGTTCCCTGGCGTACTC, Timp1-Reverse, ACCTGATCCGTCCACAAACAG; transforming growth factor (TGF)-β1-Forward, CTCCCGTGGCTTCTAGTGC, TGF-β1-Reverse, GCCTTAGTTTGGACAGGATCTG; α-SMA-Forward, GTCCCAGACATCAGGGAGTAA, α-SMA-Reverse; TCGGATACTTCAGCGTCAGGA.

### Measurement of cytokine levels by enzyme-linked immunosorbent assay (ELISA)

To determine the production and secretion of TNF-α protein in CDAA-treated mouse liver, a modified protocol that described in previous reports was used [18], [19]. In brief, a liver fragment was divided into two specimens (100 µg each). One specimen was subjected directly to protein extraction, and the amount of protein extracted was determined using a Bio-Rad protein assay kit (Bio-Rad Laboratories, Hercules, CA, USA). The other was cultured in a 24-well flat-bottomed culture plate in serum-free Dulbecco's modified Eagle's medium (D-MEM; Life Technologies) supplemented with penicillin and streptomycin (Life Technologies). After 12 hours, the supernatant was collected and the protein level measured. The amounts of TNF-α, IL6, and IL1-β proteins were measured using a Mouse ELISA Ready-SET-Go! kit (eBioscience, San Diego, CA, USA) according to the manufacturer's protocol.

### Mouse peritoneal macrophage experiments

Mouse peritoneal macrophages were isolated from 8-week-old female C57BL/6J mice. Peritoneal cells were harvested by peritoneal lavage with 10 ml PBS. Cells were re-suspended and cultured in D-MEM supplemented with 10% FCS, 100 mg/ml of penicillin, 100 mg/ml of streptomycin, and 1.25 µg/ml of amphotericin B. 1.0×10^6^ peritoneal cells were seeded into a 48-well dish, and incubated for 2 hours. Then, cells were washed in PBS, and re-cultured in the serum-free medium. To inhibit TNF-α activity, either control serum or 0.4 µg/ml of anti-TNF-α neutralizing polyclonal antibodies (R&D systems) was administered into the culture medium. After 30 minutes later, 1 µg/ml of lipopolysaccharide (LPS) were added. Medium and cells were collected 2 hours after the stimulation, and subjected to the analyses according to the methods described above.

### Statistical analyses

Results are the mean ± standard deviation unless stated otherwise. Differences between treatments, groups, and strains were analyzed using the two-tailed Student's t-test.

## Results

### *Nrd1^−/−^* mice did not develop steatohepatitis with CDAA diet

**Figure 1:**
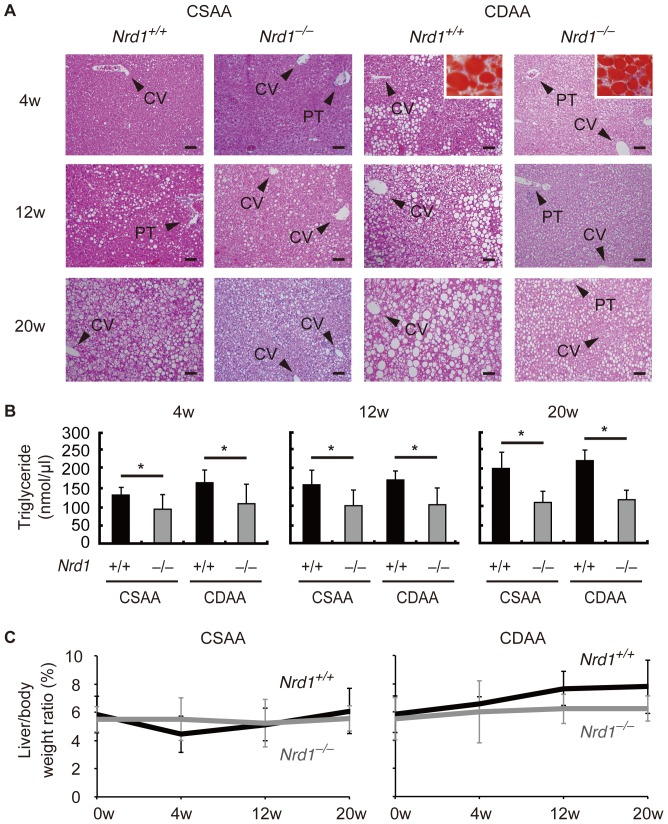
CDAA diet caused hepatic steatosis in *Nrd1^+/+^* and *Nrd1^−/−^* mice. A. Histology of the livers of *Nrd1^+/+^* and *Nrd1^−/−^* mice fed the CSAA (left) or CDAA (right) diets. Representative changes of the liver with regard to fat deposition at 4 (upper), 12 (middle), and 20 (lower) weeks during the experiments are depicted. Fat deposits were confirmed by oil red O staining shows (orange, inset). CV indicates central vein, and PT marks portal triad. Bars indicate 100 µm. B. Quantification of triglyceride in the liver. Triglyceride in the liver was increased in the livers of both *Nrd1^+/+^* and *Nrd1^−/−^* mice during administration of the CDAA or CSAA diets, although it was significantly more prominent in *Nrd1^+/+^* mice. n = 4–5, each. *P<0.05. C. There was no significant difference in the liver/body weight ratio between *Nrd1^+/+^* and *Nrd1^−/−^* mice during the experiments.

**Figure 2:**
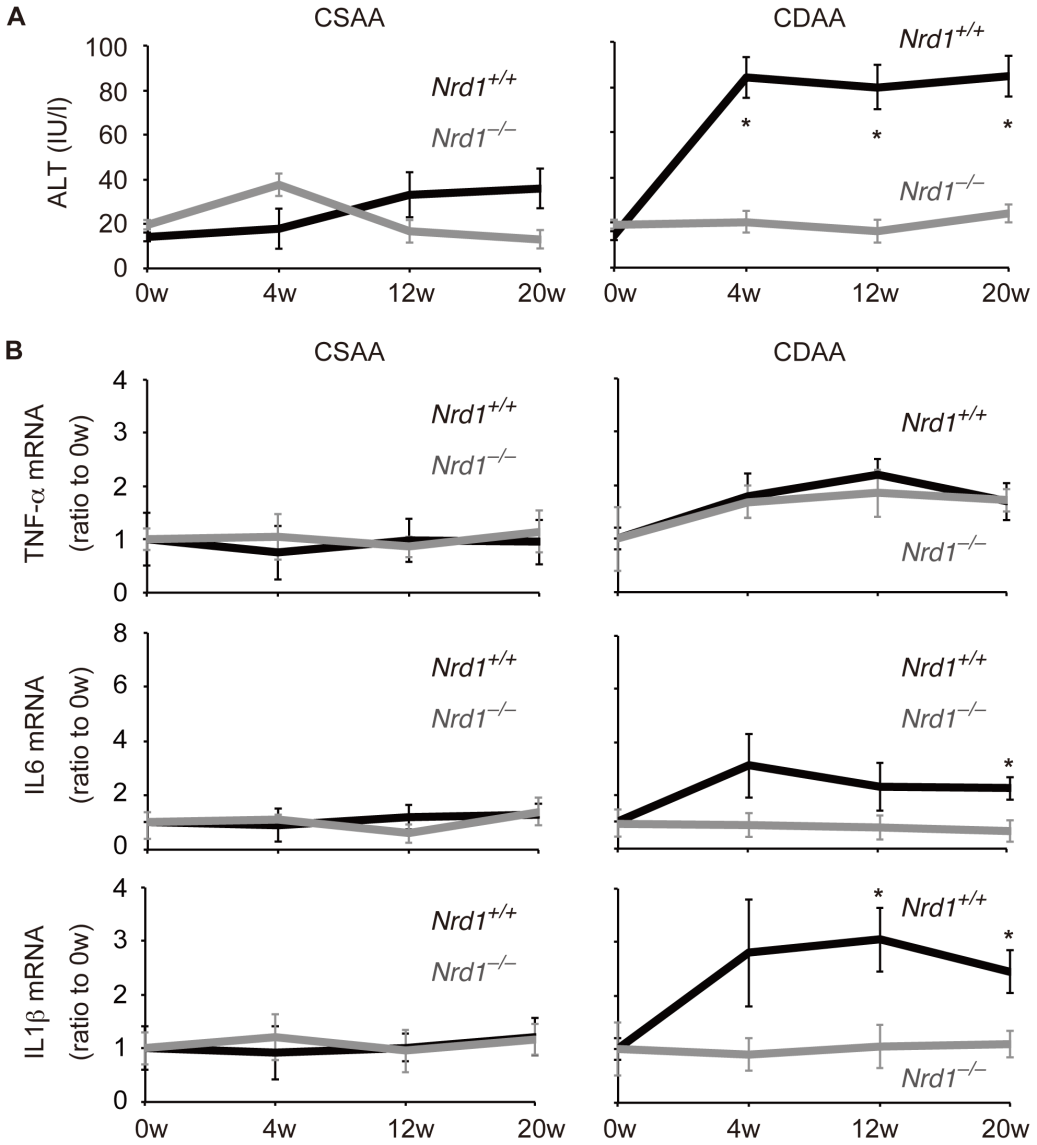
CDAA diet did not cause steatohepatitis in *Nrd1^−/−^* mice. A. Serum ALT was not elevated in both *Nrd1^+/+^* and *Nrd1^−/−^* mice fed the CSAA diet (left). Serum ALT levels were significantly elevated in *Nrd1^+/+^* mice, but were not elevated in *Nrd1^−/−^* mice upon administration of the CDAA diet (right). Each value depicts the mean ± standard errors (n = 5–15, each). *P<0.05. B. Relative expression of mRNA are shown as relative values compared to those at 0 w. mRNA of TNF-α was increased in both *Nrd1^+/+^* (significantly) and *Nrd1^−/−^* mice fed the CDAA diet compared with those fed the CSAA diet, with no significant difference between the two groups. In contrast, the mRNA expression levels of IL6 and IL1-β were increased only in *Nrd1^+/+^* mice, and these levels were significantly higher than respective values in *Nrd1^−/−^* mice fed the CDAA diet. (n = 5–16, each) *P<0.05.

The CDAA diet is deficient in choline only, but contains methionine, allowing observation of the sequential development of steatohepatitis and liver fibrotic changes in a longer experimental period in mice [4], [14]. The control CSAA diet also causes mild steatosis, but does not result in steatohepatitis and liver fibrotic changes in mice [4], [14]. To study the role of nardilysin during the development of steatohepatitis followed by liver fibrosis, *Nrd1^+/+^* and *Nrd1^−/−^* mice were fed the CSAA or CDAA diets. Histology and oil red O staining showed that fat accumulation in the livers of both *Nrd1^+/+^* and *Nrd1^−/−^* mice occurred during administration of the CDAA or CSAA diets and increased in a time-dependent manner, although fat accumulation in *Nrd1^+/+^* mice was more prominent than that in *Nrd1^−/−^* mice (Figure 1A). Size of fat deposition was greater in *Nrd1^+/+^* mice than in *Nrd1^−/−^* mice in both diet groups at each time point (Figure 1A), and triglyceride levels in the liver were significantly higher in *Nrd1^+/+^* mice (Figure 1B). There was no significant difference in the liver/body weight ratio between *Nrd1^+/+^* and *Nrd1^−/−^* mice fed CSAA or CDAA diets (Figure 1C). Thus, administration of CSAA or CDAA diets induced hepatic steatosis in mice to a varying degree. However, serum ALT levels were significantly increased in *Nrd1^+/+^* mice upon administration of the CDAA diet, whereas they were not increased in *Nrd1^−/−^* mice fed the CDAA diet (Figure 2A). Serum ALT level was elevated in neither *Nrd1^+/+^* nor *Nrd1^−/−^* mice fed the CSAA diet (Figure 2A). Consistent with these findings, qRT-PCR showed that mRNA expression of inflammatory cytokines, such as IL6 and IL1-β, was significantly increased only in *Nrd1^+/+^* mice fed the CDAA diet when fat accumulation and elevation of ALT were prominent, whereas they were not increased in *Nrd1^−/−^* mice fed the CDAA diet, and in both *Nrd1^+/+^* and *Nrd1^−/−^* mice fed the CSAA diet (Figure 2B). These data indicated that nardilysin played an important role in the development of steatohepatitis and accompanied the production of inflammatory cytokines in mice fed the CDAA diet.

### *Nrd1* was required for sufficient secretion of TNF-α

**Figure 3:**
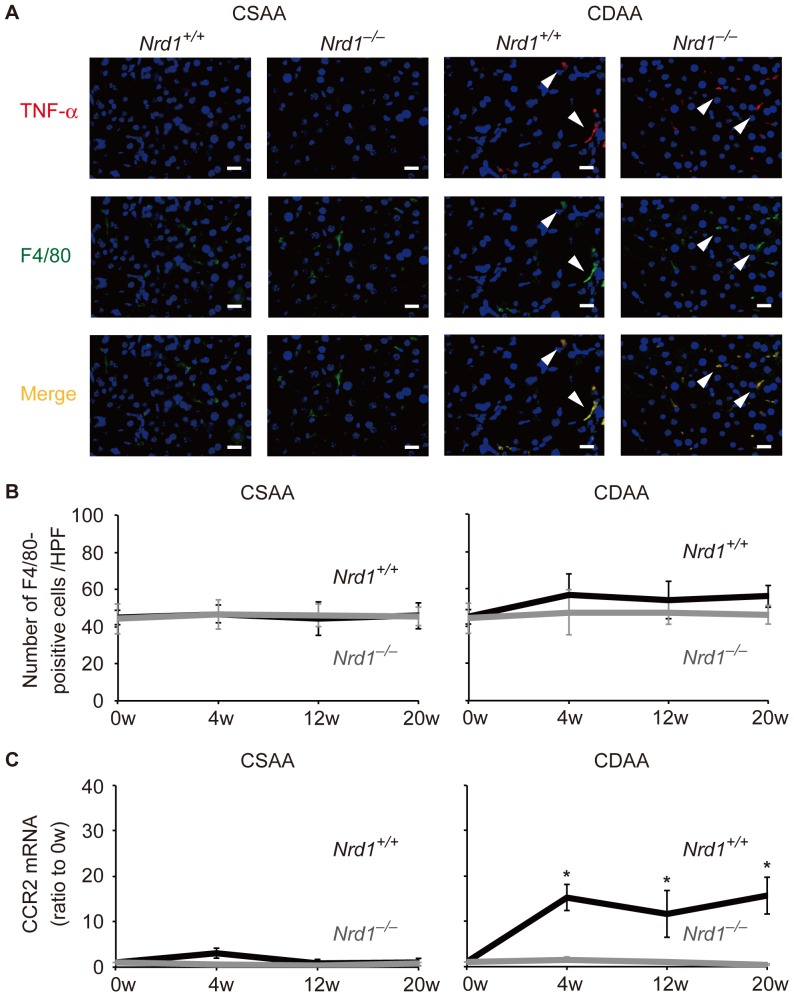
TNF-α was expressed in *Nrd1^+/+^* and *Nrd1^−/−^* mice fed the CDAA diet. A. Immunohistochemistry showed that TNF-α protein (red, arrowheads) was expressed in F4/80-positive Kupffer cells or macrophages (green, arrowheads) in the livers of both *Nrd1^+/+^* and *Nrd1^−/−^* mouse fed the CDAA diet for 20 weeks (right), but not in mice fed the CSAA diet for 20 weeks (left). A blue color indicates DAPI-positive nuclei. Bars indicate 50 µm. B. The number of F4/80-positive cells/×100 high-power field (HPF) in livers slightly increased (approximately 1.2 times) only in *Nrd1^+/+^* mice fed the CDAA diet (right). C. Relative expression of mRNA are shown as relative values compared to those at 0 w. The mRNA expression level of CCR2 was increased in *Nrd1^+/+^* mice fed the CDAA diet, and the levels were significantly higher than respective values in *Nrd1^−/−^* mice fed the CDAA diet. *P<0.05.

**Figure 4:**
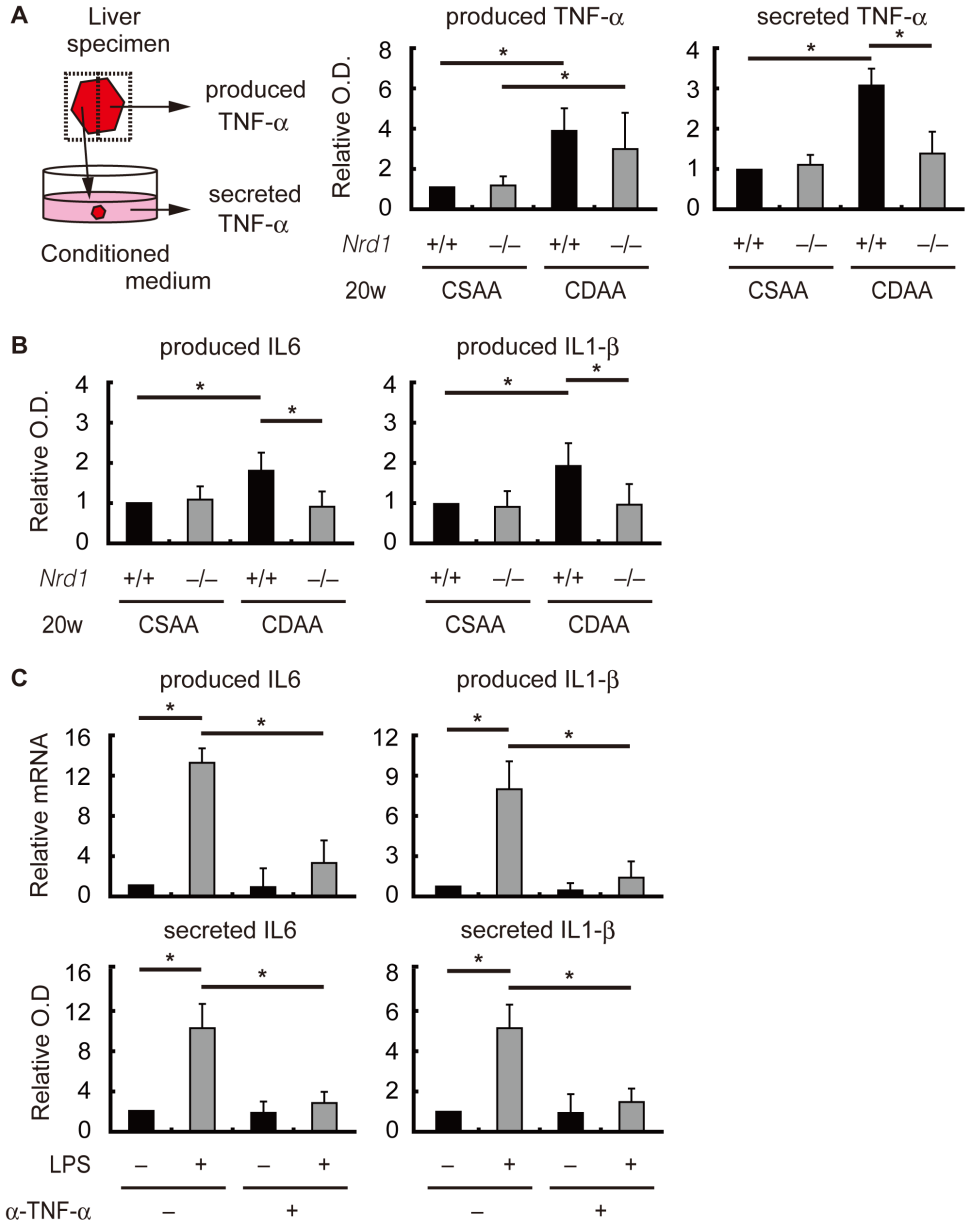
TNF-α was not sufficiently secreted from in *Nrd1^−/−^* mice fed the CDAA diet. A. A liver fragment was divided into two pieces. One was directly subjected to protein extraction directly and measurement of TNF-α (produced TNF-α). The other piece was cultured in serum-free medium for 12 hours, and the supernatant was subjected to measurement of TNF-α (secreted TNF-α). The relative optical density (O.D.) to that of *Nrd1^+/+^* fed the CSAA diet was determined by ELISA for TNF-α. Production of TNF-α from liver specimens were not significantly different between *Nrd1^+/+^* and *Nrd1^−/−^* mice fed the CDAA diet for 20 weeks (“produced TNF-α”). In contrast, TNF-α secreted from liver specimens was significantly increased in *Nrd1^+/+^* mice fed the CDAA diet for 20 weeks compared with those fed the CSAA diet; however, the elevation was not observed in *Nrd1^−/−^* mice under the same condition (“secreted TNF-α”). *P<0.05. B. Production of IL6 and IL1-β proteins were significantly increased only in *Nrd1^+/+^* mice fed the CDAA diet for 20 weeks compared with those in *Nrd1^−/−^* mice fed the CSAA diet for 20 weeks. *P<0.05. C. mRNA (upper, ‘produced’) and protein (lower, ‘secreted’) production of IL6 and IL1-β were significantly increased after LPS treatment in *Nrd1^+/+^* mouse peritoneal macrophages. However, administration of anti-TNF-α neutralizing antibodies significantly suppressed the production of IL6 and IL1-β in the presence of LPS. *P<0.05.

TNF-α is one of the key molecules that are involved in the development of NASH [4]–[7], [20]. Because secretion of activated TNF-α is the initial step in nardilysin-mediated production of inflammatory cytokines [12], we hypothesized that sufficient secretion of TNF-α by nardilysin is required for the development of steatohepatitis. Thus, we aimed to ascertain whether TNF-α was produced and secreted sufficiently in the livers of *Nrd1^+/+^* and *Nrd1^−/−^* mice fed the CDAA diet. qRT-PCR showed that the mRNA of TNF-α was increased in both *Nrd1^+/+^* and *Nrd1^−/−^* mice fed the CDAA diet, and that in contrast to the results looking at IL6 and IL1-β mRNA levels, there was no significant difference between *Nrd1^+/+^* and *Nrd1^−/−^* mice (Figure 2B). Immunohistochemistry showed that TNF-α protein was detected in F4/80-positive Kupffer cells or macrophages in both *Nrd1^+/+^* and *Nrd1^−/−^* mice fed the CDAA diet for 20 weeks (Figure 3A, right, arrowheads). Conversely, TNF-α protein was barely detected in F4/80-positive Kupffer cells or macrophages in both *Nrd1^+/+^* and *Nrd1^−/−^* mice fed the control CSAA diet for 20 weeks (Figure 3A, left). The number of F4/80-positive cells/×100 high power field (HPF) in the liver was slightly increased only in *Nrd1^+/+^* mice fed the CDAA diet but not in *Nrd1^−/−^* mice fed the CDAA diet and those in *Nrd1^+/+^* and *Nrd1^−/−^* mice fed the CSAA diet (Figure 3B). qRT-PCR showed that mRNA expression of CCR2, a recruited macrophage marker, was significantly increased in *Nrd1^+/+^* mice, but not in *Nrd1^−/−^* mice (Figure 3C). This suggested that macrophages are not sufficiently recruited in *Nrd1^−/−^* mice. At 20 weeks of a CDAA feeding, production of TNF-α protein was significantly upregulated in both *Nrd1^+/+^* and *Nrd1^−/−^* mouse livers (Figure 4A, produced TNF-α), but the increase in TNF-α protein secretion from liver specimens into the conditioned medium was decreased significantly (0.46-fold) by *Nrd1* knockout (Figure 4A, secreted TNF-α). In contrast, production of IL6 and IL1-β proteins were not increased in *Nrd1^−/−^* mice fed a CDAA diet (Figure 4B). These data suggested that nardilysin was required for the shedding of TNF-α in mice fed the CDAA diet and possibly the induction of inflammation. To further investigate that possibility, we examined whether blocking TNF-α suppresses the production of IL6 and IL1-β. We used *Nrd1^+/+^* mouse peritoneal macrophages as substitutes for Kupffer cells and recruited macrophages in the liver, and examined the effect of pre-incubation with anti-TNF-α neutralizing antibodies on the production of IL6 and IL1-β. Following LPS stimulation mRNAs and secreted proteins of both IL6 and IL1-β from macrophages were significantly increased, and administration of anti-TNF-α neutralizing antibodies significantly suppressed the production of IL6 and IL1-β (Figure 4C). This also suggested that TNF-α secretion played an important role to induce IL6 and IL1-β production in mice.

### *Nrd1^−/−^* mice were resistant to CDAA diet-induced liver fibrotic changes

**Figure 5:**
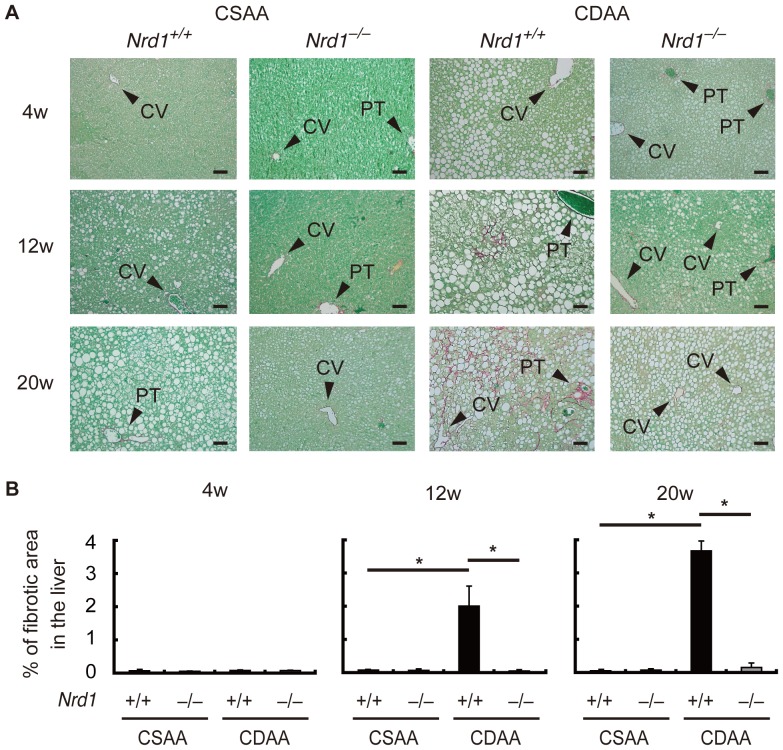
Liver fibrotic area was not observed in *Nrd1^−/−^* mice fed the CDAA diet. A. Liver fibrosis was determined by Sirius red staining (red) in *Nrd1^+/+^* and *Nrd1^−/−^* mice at 4 (upper), 12 (middle), and 20 (lower) weeks in the livers of *Nrd1^+/+^* and *Nrd1^−/−^* mice fed the CSAA or CDAA diet. Fibrotic changes were not observed in *Nrd1^+/+^* or *Nrd1^−/−^* mice fed the CSAA diet (left). Fibrotic changes were prominent in *Nrd1^+/+^* mice, but not in *Nrd1^−/−^* mice fed the CDAA diet (right). Bars indicate 100 µm. B. Quantification of fibrotic areas. Fibrotic areas was observed and increased in a time-dependent manner only in the livers of *Nrd1^+/+^* mice fed the CDAA diet. n = 5–8, each. *P<0.05.

**Figure 6:**
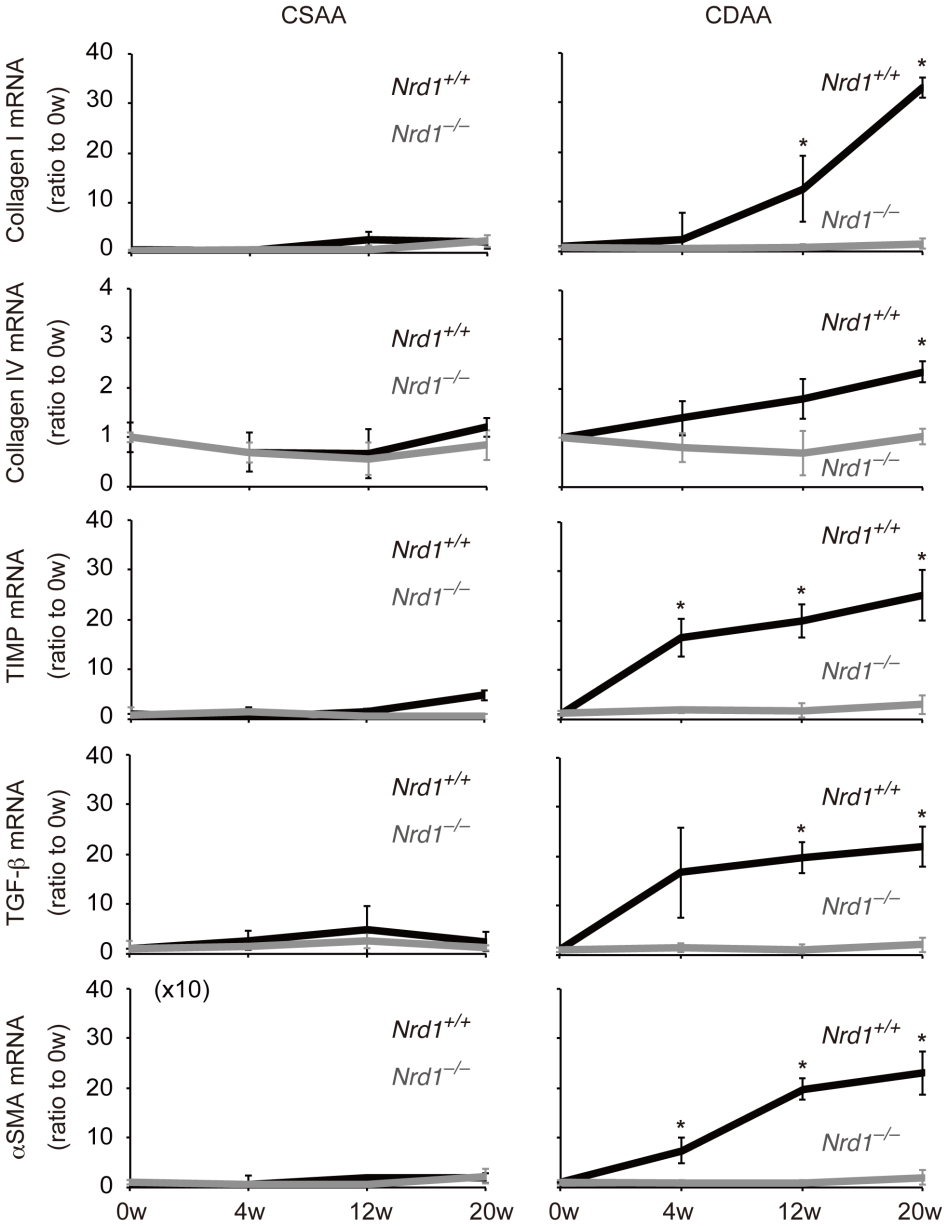
Fibrogenic factors were not elevated in *Nrd1^−/−^* mice fed the CDAA diet. During CDAA diet administration, mRNA expression levels of collagen I, collagen IV, TIMP, TGF-β, and αSMA were significantly increased in the livers of *Nrd1^+/+^* mice but not in those of *Nrd1^−/−^* mice. Those factors were not altered by administration of the CSAA diet in *Nrd1^+/+^* or *Nrd1^−/−^* mice. *P<0.05.

**Figure 7:**
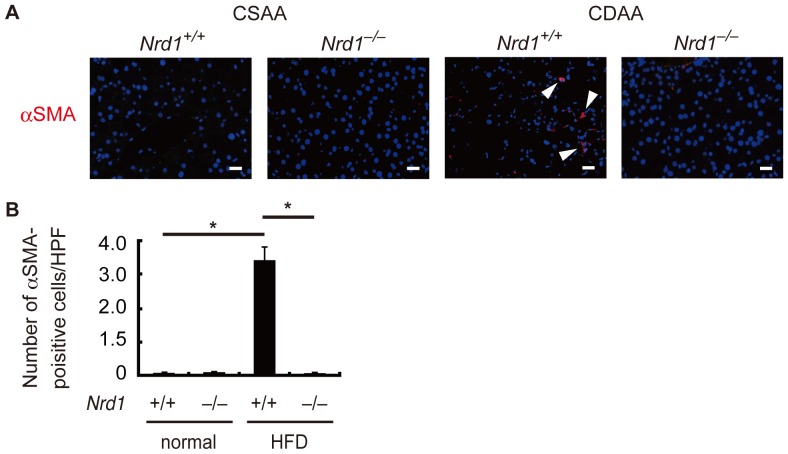
Activated myofibroblasts were not observed in *Nrd1^−/−^* mice fed the CDAA diet. A. Immunostainings for αSMA demonstrated that activated myofibroblasts were detected in *Nrd1^+/+^* mice fed a CDAA diet for 20 weeks, but not in *Nrd1^+/+^* mice. Activated myofibroblasts were hardly detected by administration of the CSAA diet in *Nrd1^+/+^* or *Nrd1^−/−^* mice. Bars indicate 100 µm. B. The number of αSMA-positive cells/×400 high-power field (HPF) in livers increased only in *Nrd1^+/+^* mice fed the CDAA diet for 20 weeks. *P<0.05.

Persistent steatohepatitis results in hepatic fibrosis [1]–[4]. Using Sirius red staining we investigated whether secretion/production of inflammatory cytokines enhanced by nardilysin was associated with the development of liver fibrotic changes. Four weeks after CDAA feeding, fibrotic changes were not prominent in both *Nrd1^+/+^* and *Nrd1^−/−^* mice (Figure 5A and B). Twelve weeks after CDAA feeding, fibrotic changes were observed in *Nrd1^+/+^* mice, whereas such changes were not prominent in *Nrd1^−/−^* mice (Figure 5A and B). At 20 weeks of CDAA diet administration, fibrotic changes in *Nrd1^+/+^* mice became more prominent, while they were not observed in *Nrd1^−/−^* mice (Figure 5A and B). Fibrotic changes were not observed throughout the experiments in both *Nrd1^+/+^* and *Nrd1^−/−^* mice fed a CSAA diet (Figure 5A and B). Consistently, the increased mRNA expression of fibrogenic markers such as collagen I, collagen IV, TIMP1, TGF-β, and αSMA in *Nrd1^+/+^* mouse livers were not observed in *Nrd1^−/−^* mice fed the CDAA diet (Figure 6). Immunostainings for αSMA demonstrated that activated myofibroblasts were detectable only in *Nrd1^+/+^* mice fed a CDAA diet (Figure 7A and B). Thus, nardilysin played a pivotal role in the development of liver fibrosis caused by the CDAA diet.

### *Nrd1^−/−^* mice were resistant to high fat diet-induced liver fibrogenesis

**Figure 8:**
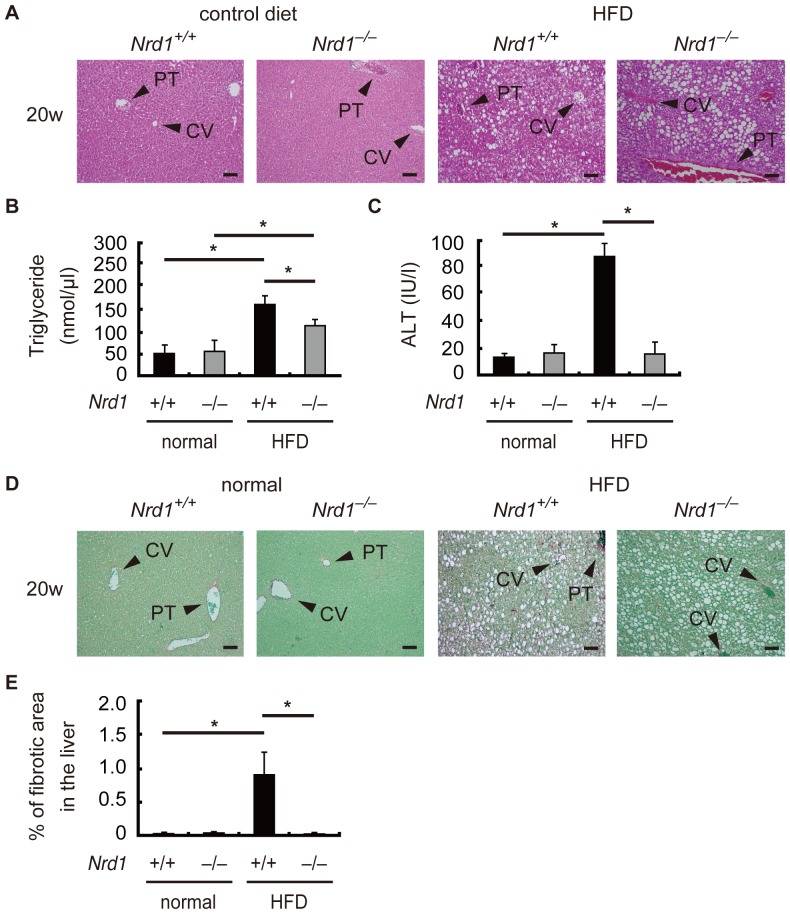
Liver fibrogenesis was not observed in *Nrd1^−/−^* mice fed the HFD. A. Steatosis was observed in both *Nrd1^+/+^* and *Nrd1^–/–^* mice after 20-week HFD administration (right), but not in those fed a normal control diet (left). Bars indicate 100 µm. B. Quantification of triglyceride in the liver. Triglyceride was elevated in the livers of both *Nrd1^+/+^* and *Nrd1^–/–^* mice after 20-week HFD administration, although it was significantly higher in *Nrd1^+/+^* mice. n = 4, each. *P<0.05. C. Serum ALT levels were significantly elevated in *Nrd1^+/+^* mice upon administration of the HFD, but were not elevated in other mouse groups. *P<0.05. D. Fibrotic area was less prominent in *Nrd1^−/−^* mice than in *Nrd1^+/+^* mice (right). Bars indicate 100 µm. E. Fibrotic area was observed only in the livers of *Nrd1^+/+^* mice fed the HFD (right). n = 5, each. *P<0.05.

**Figure 9:**
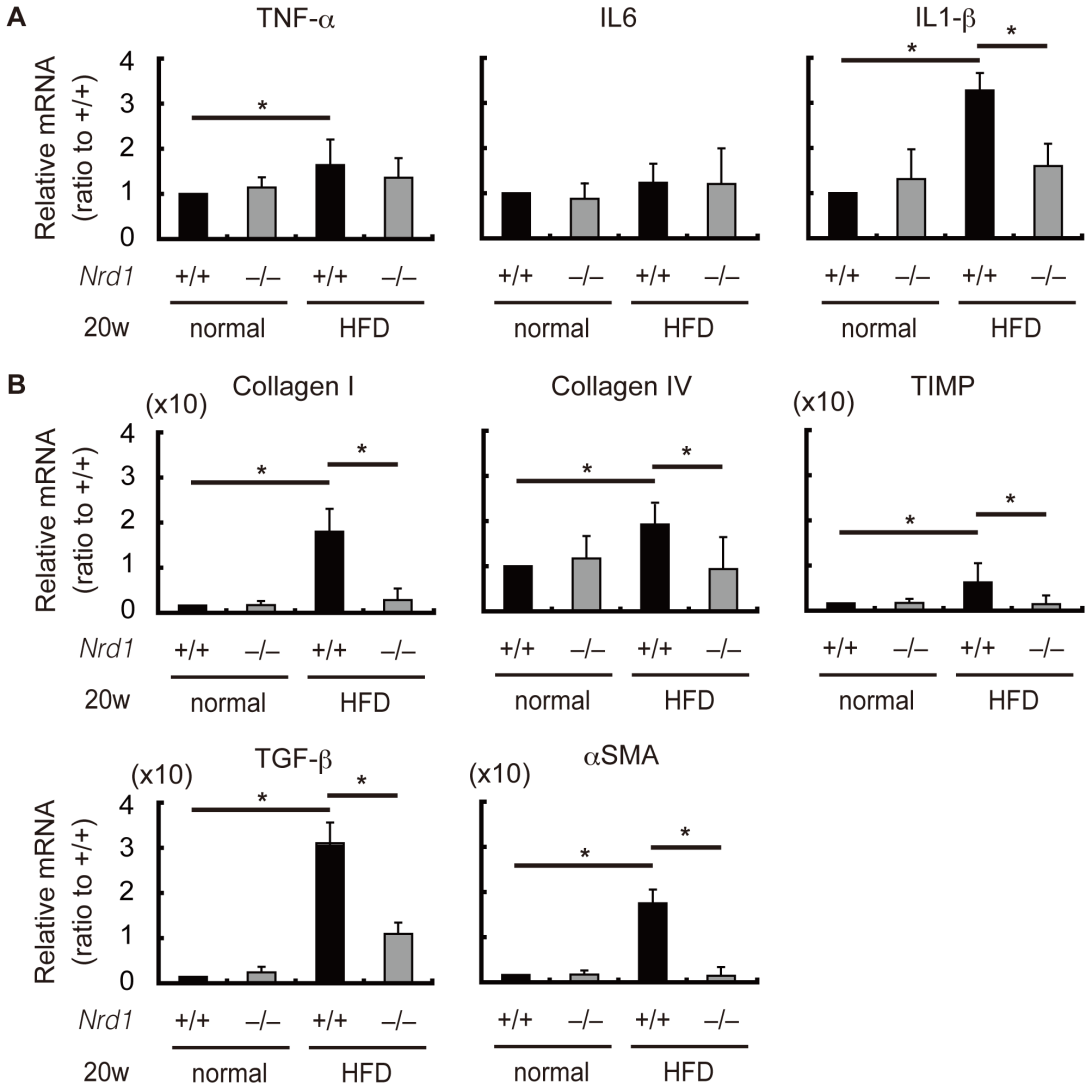
Inflammatory and fibrogenic factors were not increased in *Nrd1^−/−^* mice fed the HFD. A. mRNA of TNF-α was slightly increased in both *Nrd1^+/+^* (significantly) and *Nrd1^−/−^* mice. In contrast to *Nrd1^+/+^* mice, the mRNA expression level of IL1-β was not increased in *Nrd1^−/−^* mice. *P<0.05. B. mRNA expression levels of collagen I, collagen IV, TIMP, TGF-β, and αSMA in the livers of *Nrd1^+/+^* mice fed a HFD for 20 weeks were significantly higher than the respective values of *Nrd1^+/+^* mice fed the control diet. However, they were not altered by HFD in *Nrd1^−/−^* mice. *P<0.05.

To further confirm the role of nardilysin in the development of steatohepatitis followed by liver fibrotic changes, *Nrd1^+/+^* and *Nrd1^−/−^* mice were also fed HFD. Similar to the CDAA diet, HFD administration for 20 weeks induces hepatic steatosis and liver fibrogenesis [16]. In the present study, steatosis was observed more prominently in *Nrd1^+/+^* mice compared to *Nrd1^−/−^* mice at 20 weeks of HFD administration, but not in mice fed a normal control diet (Figure 8A). Consistently, triglyceride in the liver were elevated in *Nrd1^+/+^* and *Nrd1^−/−^* mice (Figure 8B). However, serum ALT levels were significantly increased in *Nrd1^+/+^* mice upon 20-week administration of the HFD, whereas they were not increased in *Nrd1^−/−^* mice fed the HFD (Figure 8C). Furthermore, fibrotic changes were detected only in *Nrd1^+/+^* mice fed a HFD (Figure 8D and E). Consistent with this finding, qRT-PCR showed that the mRNA expression of IL1-β was significantly increased only in *Nrd1^+/+^* mice at 20 weeks of HFD feeding, but not in that of *Nrd1^−/−^* mice (Figure 9A). mRNA expression levels of collagen I, collagen IV, TIMP, TGF-β, and αSMA were significantly increased in the livers of *Nrd1^+/+^* mice fed a HFD for 20 weeks, but not in those of *Nrd1^−/−^* mice (Figure 9B). Therefore, nardilysin also played an important role in the development of steatohepatitis and liver fibrogenesis induced by HFD in mice.

## Discussion

In the present study, we demonstrated that steatosis was induced by the CDAA diet in both *Nrd1^+/+^* and *Nrd1^−/−^* mice, although fatty changes were less prominent in *Nrd1^−/−^* mice. Importantly, steatohepatitis followed by liver fibrotic changes was observed only in *Nrd1^+/+^* mice and not in *Nrd1^−/−^* mice. Secretion of TNF-α, and the production of inflammatory cytokines and fibrogenic factors were not upregulated in *Nrd1^−/−^* mice as compared with *Nrd1^+/+^* mice. In the HFD model, steatohepatitis and liver fibrogenesis were hardly observed in *Nrd1^−/−^* mice. These data suggested that nardilysin plays an important role in the development of steatohepatitis followed by liver fibrosis.

In mice fed with the CDAA diet, the levels of hepatic triglyceride content were lower in *Nrd1^−/−^* mice compared with those in *Nrd1^+/+^* mice, suggesting the possibility that nardilysin is involved in the regulation of hepatic lipid synthesis. A decreased steatosis in *Nrd1^−/−^* mice may partly affect hepatic inflammation. However, steatosis did occur in the liver of *Nrd1^−/−^* mice; on the other hand, hepatic inflammation was not observed despite the presence of steatosis in *Nrd1^−/−^* mice. This indicated that nardilysin has an important role in the initiation and/or promotion of inflammatory responses induced by the CDAA diet. Persistent inflammation distinguishes steatohepatitis from simple hepatic steatosis [1]–[3]. Among pro-inflammatory factors, TNF-α is one of the key molecules that initiate inflammatory cascades, and its role in the progression of NASH has been discussed [4]–[7]. For example, apoptotic change in the liver, which contributes to the progression of NASH, is inhibited by an anti-TNF receptor neutralizing antibody or pentoxifylline in a mouse model of NASH [20]. The absence of TNFR1, a receptor for TNF-α, reduces IL6 mRNA production in the liver fed with the HFD even in the presence of elevated serum TNF-α [21]. The absence of TNFR1 also reduces liver lipid accumulation and macrophage accumulation in livers of HFD-fed mice [21]. Thus, inhibition of TNF-α signaling appears to plays a pivotal role to suppress inflammatory reactions in NASH as well as other inflammatory disorders [22]. Although clinical application of anti-TNF-α therapy has not been established in the treatment of human NASH, anti-TNF-α neutralizing antibodies are effectively used to treat various human inflammatory disorders, such as rheumatoid arthritis and inflammatory bowel diseases [6], [7]. We previously reported that nardilysin is essential for the sufficient activation of TNF-α in cooperation with TACE [10]–[12]. By the knockdown of *Nrd1*, TNF-α secretion is decreased concomitantly with decreased TACE activity, and the production of inflammatory cytokines such as IL6 and IL1-β is significantly suppressed [10]–[12]. In the present study, it is worth noting that TNF-α secretion from liver specimens was decreased significantly in *Nrd1^−/−^* mice fed the CDAA diet, while TNF-α production was not different between *Nrd1^+/+^* and *Nrd1^−/−^* mice fed the CDAA diet. Consistently, the production of various inflammatory cytokines were not increased in the livers of *Nrd1^−/−^* mice. Although the precise mechanism of the decreased inflammatory responses in *Nrd1^−/−^* mice was not clear, it appeared likely that the impaired release of TNF-α in *Nrd1^−/−^* mouse livers was one of the reasons for the reduced inflammatory reactions in *Nrd1^−/−^* mice. As well, impaired recruitment of macrophages into the liver may also contribute to the reduced inflammatory reactions in *Nrd1^−/−^* mice. It would be also possible that different activation status of TNF-α and inflammatory responses conversely affect difference of fatty contents between *Nrd1^+/+^* and *Nrd1^−/−^* mice. Whatever the case, nardilysin seemed to play an important role in the development of steatohepatitis and liver fibrosis presumably through TNF-α activation.

Previous studies have shown that Kupffer cells and recruited macrophages interact with hepatic stellate cells, accelerate their activation, and promote the fibrogenic responses [4], [17]. Activated myofibroblasts also promote the remodeling of the extracellular matrix and contribute to liver fibrosis [5]. Indeed, our immunohistochemical analyses showed that Kupffer cells and macrophages were major producers of TNF-α in the livers of mice fed the CDAA diet, and that αSMA-positive myofibroblasts were not prominent in *Nrd1^−/−^* mice. Decreased release of TNF-α from Kupffer cells and recruited macrophages could be one of the mechanisms for the suppression of diet-induced steatohepatitis in *Nrd1^−/−^* mice, and thus nardilysin in Kupffer cells and recruited macrophages may be required for the progression of NASH and liver fibrosis, concomitantly with the recruitment of myofibroblasts. However, we could not completely exclude the possible contribution of nardilysin in other cells such as hepatocytes or endothelial cells for the development of NASH and liver fibrosis. Therefore, genetically-engineered mice lacking or strongly expressing nardilysin in Kupffer cells and macrophages may be required to confirm our hypothesis in future studies.

In summary, the present study indicates that nardilysin contributes to the development of diet-induced NASH and liver fibrotic changes by regulating chronic liver inflammation. Nardilysin could be an attractive target for anti-inflammatory therapy against NASH and liver fibrosis.
